# Healthcare professionals’ perceptions of impacts of the Covid-19-pandemic on outpatient care in rural areas: a qualitative study

**DOI:** 10.1186/s12913-021-07261-y

**Published:** 2021-12-02

**Authors:** Madlen Hoerold, Marc Gottschalk, Carla Maria Debbeler, Heike Heytens, Saskia Ehrentreich, Ruediger C. Braun-Dullaeus, Christian Apfelbacher

**Affiliations:** 1 Institute of Social Medicine and Health Systems Research, Medical Faculty, Otto von Guericke Universität Magdeburg, Leipziger Str. 44, 39120 Magdeburg, Germany; 2 University Clinic for Cardiology and Angiology, Medical Faculty, Otto von Guericke University, Leipziger Str. 44, 39120 Magdeburg, Germany

**Keywords:** Family physicians, Cardiologists, Healthcare, Content analysis, Germany

## Abstract

**Background:**

Measures to manage the COVID-19 pandemic have led to impacts on healthcare systems and providers worldwide. Outpatient healthcare professionals (HCPs) provide the majority of patient care. Insight into their experiences during a pandemic is rare. Therefore, we explored how primary and secondary care HCPs in a rural area in Germany experienced their work during the pandemic and what health-related outcomes they perceived in their patients. In this context, we also examined the impact on access to and utilization of healthcare and working conditions.

**Methods:**

We conducted a qualitative interview study with outpatient HCPs. We recruited by e-mail, telephone, professional networks and personal contacts. Data were collected between August 2020 and January 2021. All interviews were audio recorded, transcribed, and analysed using qualitative content analysis.

**Results:**

Our sample consisted of 28 HCPs (15 family physicians, 7 cardiologists, and 6 non-physician assistants, 12 female) from Saxony-Anhalt, Germany. HCPs experienced fewer consultations as well as cancellations by hospitals and secondary care physicians, especially at the beginning of the Covid-19-pandemic, while they continued throughout to provide outpatient care. They quickly adopted changes in practice organisation and healthcare provision. There was a shift towards telephone consultations, home visits as well as unconventional consultations e.g. through the practice window. Family physicians used personal relationships to support utilization of healthcare and to avoid health-related effects. Social tension and burden seemed to interact with a perceived lack of preparedness, the pandemic-related changes in their working condition as well as access to and utilization of healthcare. Chronic disease monitoring was postponed, which could have consequences in the course of disease of patients. HCPs experienced effects on patients’ psychological well-being.

**Conclusion:**

Our study demonstrates the impacts of Covid-19-pandemic on outpatient care in rural areas and emphasizes its importance. HCPs experienced impacts on access to and utilization of healthcare, working conditions and health-related outcomes. Health policy should create a framework for healthcare to support outpatient care in rural areas with a looming undersupply of primary and secondary care in order to maintain healthcare and reduce pandemic impacts.

**Supplementary Information:**

The online version contains supplementary material available at 10.1186/s12913-021-07261-y.

## Background

Starting in March 2020, Germany and other countries implemented non-pharmaceutical intervention measures to reduce the spread of SARS-CoV-2 infections and avoid an overburdening of the healthcare system [1]. Shortly after, the German Society for General and Family Medicine published its S1 treatment recommendation “New Coronavirus (SARS-CoV-2) - Information for Family Practice” [2]. Furthermore, there were adjustments in all areas of medical care [3] with hospitals being the focus of attention. For example, there was an expansion of inpatient, especially intensive care treatment capacities for patients with severe COVID-19, combined with the request to postpone elective (postponable) surgeries and interventions until further notice [4]. Nearly 94% of PCR tests for suspected potential COVID-19 infection in the first quarter of 2020 in Germany were provided in an outpatient setting. The vast majority of COVID-19 patients in Germany have been able to receive care in an outpatient setting to date [5]. This has also prevented hospital overload, as occurred in Italy and should not be underestimated [6]. Rural areas in Germany were already facing multiple challenges in healthcare distribution before the COVID-19 pandemic. Nationwide provision of medical care will be threatened in the future by distinct shifts between urban and rural areas, by societal transformation of the medical profession, poorer access to physicians’ facilities and a growing number of older people in rural areas [7]. The federal state Saxony-Anhalt (Germany) is mainly rural and faces with a shortage of outpatient physicians. Thus, medical care will become more difficult in the future, especially in rural areas. Currently, nearly 300 primary care physician vacancies. More than 150 family physicians and almost 200 of the outpatient secondary care physicians have already passed the age of 65 [8]. Overall 2,180,684 inhabitants live on 20,454 km^2^ (106.7 inhabitants per square kilometer) [9]. The population is overaged. In 2020, the share of the population aged 65 and older in the total population in Saxony-Anhalt was around 27.4%. This compares with 22% in Germany [10]. Furthermore, Saxony-Anhalt has the highest prevalence rates of chronic diseases (ischemic heart disease, diabetes, hypertension and heart failure) in Germany [11–14], and thus many parts of the population are at high risk for a severe course of COVID-19 disease [15, 16]. At the beginning of the pandemic in Germany, Saxony-Anhalt was less affected compared to other German federal states. In 2020, 30,933 COVID-19 disease cases (1418.5 cases per 100,000 inhabitants) and 602 COVID-19 related deaths were registered. The number of reported COVID-19 disease cases is currently 99,752 (status: August 9, 2021) [17]. Until August 2021 (status: August 09, 2021), 158 COVID-19 related deaths per 100,000 inhabitants were documented in Saxony-Anhalt. This compares to 110 COVID-19 related deaths per 100,000 inhabitants in Germany (total) [18].

Insights into the experiences of outpatient healthcare professionals (HCPs) during the COVID-19-pandemic is sparse. With rural healthcare already facing challenges in Germany, it is crucial to obtain insights into how the COVID-19 pandemic has affected outpatient primary and secondary care. In our study, we used Saxony-Anhalt as an example to examine how outpatient HCPs in a rural area in Germany experienced their work during the pandemic and what health-related outcomes they perceived in their patients. In this context, we also examined the impact on access to and utilization of healthcare as well as working conditions. These findings can be used to strengthen rural healthcare during the current pandemic as well as for future challenges.

## Methods

### Study design

A qualitative study with a structuring content analysis approach [19] was used to obtain insights into how outpatient HCPs in a rural area in Germany experienced their work during the COVID-19-pandemic. The study was embedded in our project “KARLA - KARdiologische LandAssistenz” (Cardiology support in rural areas). The project belongs to the research network, autonomy in old age “funded by the European Regional Development Fund (ERDF) of the European Union and the ministry of economics, research and digitalisation of Saxony-Anhalt, Germany (ZS/2018/12/96167). Study activities were all conducted in German. The local ethics committee of the University Hospital Magdeburg (70/20) approved this study.

### Participants

For the current study, we recruited outpatient healthcare professionals in Saxony-Anhalt, a federal state of Germany with a mainly rural character. With regard to the “KARLA” project, we included healthcare professionals with special interest or responsibility in relation to cardiological care, e.g. outpatient cardiologists, family physicians (FP) and non-physician assistants in primary care offices in Saxony-Anhalt. We excluded healthcare professionals who did not work in Saxony-Anhalt and/or were not involved in outpatient cardiology care. Another reason for exclusion was lack of written informed consent. We contacted 69 family physicians, 25 secondary care physicians (cardiologists), and 12 non-physician assistants in Saxony-Anhalt by e-mail, telephone, professional networks, and personal contact, in order to reach heterogeneity in terms of individual characteristics, regions and outpatient care. We used the publicly available contact information of the medical practices for contact. Seventy-six contacts actively declined to participate in the study due to lack of interest or time, or did not respond after initial contact. There were two drop-outs of participants between recruitment and the interview. One participant had consented to the interview, however, not to the data recording and analysis, and one participant had different expectations regarding the time and methodological approach. All participants received a written informed consent form before we made an appointment for the respective interview. Signed written informed consent forms from all participants are available. Overall, 28 HCPs were recruited to participate in an interview. Patients or the public were not involved in recruiting. All study activities were conducted in accordance with the declaration of Helsinki [20] and in compliance with the relevant legal regulations.

### Data collection

We developed a semi-structured thematic interview guide to investigate the impacts of the COVID-19-pandemic on outpatient care, working conditions, and patients’ health-related outcomes in rural areas during the first year of the pandemic. The interview guide was discussed and reviewed together with the project leaders. We conducted two interviews as pilot and included them in the sample.

We asked the following questions:

- How do you experience your work during the COVID 19 pandemic?
- For what reasons have, your patients visited your practice in recent weeks.
- How are you currently manage to get reliable information?
- Which health effects do you perceive in your patients due to COVID-19?

In addition, we explored the provision of services and special needs of patients with chronic heart diseases in outpatient care (Supplementary material). The results of these questions are not considered in the following.

Because of the COVID-19 related restrictions, interviews were conducted via telephone. The semi-structured approach supported the interviewers to ensure comparable conditions during data collection. We used fieldnotes to collect socio-demographic data (sex, age, place/region of work) and information on professional biographies (medical specialisation).

The interviewers were female with a varying degree of experiences in conducting qualitative research, however prior experiences in healthcare (one nurse, one paramedic). The coding and interpretation team consisted of five researchers, with varying levels of prior experience in conducting qualitative interview studies. We informed the participants about the professional background of the interviewers, the aim of the research and again about the relevant data protection and privacy issues before the interview. Data collection occurred until data saturation, which was reached when no new topics appeared after three consecutive interviews.

### Data processing, analysis and reporting

The data (mp3-files) were transcribed verbatim [21, 22] and pseudonymized. First, we familiarized ourselves with the material and read all the transcripts several times. For data analysis we chose the qualitative structuring content analysis according to Mayring [19–24]. A deductive coding guide was used to analyse the textual material. This technique involves the theory-driven construction of a coding guideline, consisting of category definitions, anchor examples, and coding rules [23]. We collaboratively developed a coding guide with specific definitions and coding rules based on previous studies and our thematic interview guide. Two researchers (MH/CD) independently coded all transcripts. In addition, anchor examples were included from the interviews (Table 1). For each interview, we assigned every text passage that referred to one of the categories in the coding guide to the appropriate category. Text components that did not contribute to the content were not edited, and content overlaps in the text were coded several times. We used MAXQDA [37] for data management and coding. During the process of categorising the interviews, further sub-categories were added inductively, for example when a participant mentioned individual features of the working conditions. According to Mayring [24], this deductive/inductive procedure is suitable when themes to be analysed are fixed in advance (e.g. by a thematic interview guide), but the material per theme should be reduced. We developed anchor examples and coding definitions for the inductive sub-categories to achieve a more transparent coding process. We performed a communicative validation of the inductively formed subcategories in collaborative research and interpretation work [38] via a video conferencing system (MH, HH, CD, SE, MG). We started by coding in German and then translated into English. We did not perform respondent validation. Reporting of this study is based on the COREQ Checklist [39].

**Table 1:** Deductive coding guideline

Category definition	Anchor Examples	Coding Rules
**Access to and utilization of healthcare** [25–27] Pertains to the perceived impact of the Covid-19-pandemic on access to and utilization of primary and secondary care in rural areas.	"We also had many patients who did not come to the medical office during this time." (Martina Stünznagel, family physician)	Clear assignment; Multiple responses allowed
**Working conditions** [28–32] Pertains to the perceived impact of the Covid-19-pandemic on working condition of HCPs in primary and secondary care in rural areas.	"I did not work less, rather more; I had the feeling, and then tried to care for the patients in a way that was appropriate under the circumstances." (Georg Hassel, cardiologist)	Clear assignment; Multiple responses allowed
**Health-related outcomes** [33–36] Pertains to the perceived impact of the Covid-19-pandemic on health-related outcome in patients in rural areas.	"[…] that has reduced the medical care, the closeness of the medical care and thus with sufficient probability also, I say, the prognosis worsened, that will already be so." (Franz Schulze, cardiologist)	Clear assignment; Multiple responses allowed

## Results

### Study sample

The study sample consisted of 28 healthcare professionals: 15 family physicians, seven cardiologists, and six non-physician assistants from Saxony-Anhalt, Germany. 42,8 % were female. All participants worked in outpatient medical care and had a mean professional outpatient experience of 14.0 ± 11.6 years. Further details on the characteristics of the participants are shown in Table 2. We conducted the interviews between August 2020 and January 2021. They varied in duration from 9:20–56:24 min (mean 21:40 ± 8:57 min).

**Table 2:** Characteristics of the study sample

	Family physician	Cardiologist	Non-physician assistant	Entire Sample
N	15 (5 female)	7 (1 female)	6 (6 female)	28 (12 female)
Female (in %)	33%	14.3%	100%	42.9%
Age (MD ± SD)	52.8 ± 10.37	50.3 ± 6.5	39.2 ± 5.67	49.3 ± 10.19
Region (inhabitants per square kilometer)				
< 100 inhabitants per square kilometer	11	1	3	15
100–500 inhabitants per square kilometer	4	4	3	11
> 500 inhabitants per square kilometer	0	2	0	2
Interview duration (MD in min. ± SD)	18:53 ± 05:16	25:05 ± 13:08	24:12 ± 07:31	21:40 ± 08:57
Years of outpatient experience (MIN-MAX; MD ± SD)	1–47 19.42 ± 13.19	1–21 6.71 ± 7.23	2–28 8.8 ± 7.05	1–47 13.96 ± 12.15

 In the following, we report our findings – the three main categories and corresponding sub-categories - supplemented with a definition of the sub-categories at the beginning.

### Access to and utilization of healthcare

The Covid-19-pandemic induced impacts on “***Access to and utilization of healthcare”*.** The four sub-categories along with brief definition and anchor example presented in Table 3:

**Table 3:** Access to and utilization of health care **-** sub-categories, brief definition and anchor example

Sub-category	Sub-categroy definition	Anchor example
Fewer consultations	Pertains to the reduction of medical consultations.	“In the second quarter, fewer number of cases and significantly fewer patient contacts.” (Hartmut Gendermann, family physician)
Cancellations	Pertains to cancellations of appointments, examinations, and surgeries, both by patients and healthcare professionals.	“[...] when the topic was quite topical. March, April, into May, many appointments, check-ups were canceled. Even, surgeries canceled, diagnostic procedures canceled." (Christoph Zobel, family physician)
Availability	Pertains to the experiences of always being available for patients.	“Due to the fact that the physicians’ offices, I must speak for all colleagues here, were always available during the entire Corona period and also for the [...] care of the patients, even though many tried to solve the question at the window and through the window and otherwise a lot with protective measures, all patients nevertheless got their medication and also the most important examinations that were necessary.” (Volker Heinze, family physician)
Normality returned	Pertains to the experiences that normality has returned regarding access and utilization of healthcare.	“That is currently no longer the case here. That means we have the same number of patients. No cancellations-. As they are scheduled, they come.” (Michael Haase, cardiologist)

#### Fewer consultations

HCPs reported *“[ …] the first months [ …] fewer, significantly fewer patients, so that we also had to schedule our workforce differently. We had almost no patients in the first weeks and I assume that many-, with problems simply did not come and sat it out at home” (Marion Stittich; family physician)*. Cardiologists less often reported a decrease in utilization. Since patients in rural areas sometimes wait for a specialist appointment for a very long time, these *"[...] appointments also (seemed to be) perceived as particularly valuable" (Christian Schneider, cardiologist).*

#### Cancellations

Participants voiced that hospitals and medical specialists reduced or stopped (elective) treatments. This led to delays in diagnostics and therapy combined with a prolonged need for post-discharge outpatient treatment. The cancellation of healthcare services by medical specialists and hospitals and, at the same time, the feeling of securing outpatient healthcare caused astonishment among some participants:

*“The thing that has bothered me in Corona now, is that so much has been shut down, yes. The hospitals did not do many things. Colleagues, professional colleagues did not do many things, because they said, oh I’ m so close. Yeah, my God, I am close every day" (Beate Beyer, family physician).*

Furthermore, patients cancelled appointments, examinations, and surgeries due to fear and insecurity.

#### Availability

Family physicians and cardiologists described how they provided access to healthcare, especially for non-COVID-19 patients. They explained that they “*[...] continued throughout”.* For the *“[…] patients who hesitated to come, who then had an appointment during the lockdown and said, [...], it is too uncertain for me and I’m afraid, [...]" (Sabine Emrich, family physician)* individual solutions were sought*.*

#### Normality returned

Participants’ experienced changes in access and utilization over the first year of the pandemic. After reduced access to and utilization of healthcare at the beginning of the pandemic, HCPs reported that they got *“almost back to normal operations" (Carsten Melz, cardiologist).* The number of cancellations had reduced, and patients are again visiting doctors’ offices more frequently. *“People continue [ …] to have the same problems as before" (Christoph Zobel, cardiologist)*.

### Working conditions

This category included experiences on impacts on working conditions of HCPs in outpatient care, which we represented in six sub-categories. The sub-categories along with brief definitions and anchor examples are presented in Table 4:

**Table 4:** Working condition **-** sub-categories, brief definition and anchor example

Sub-category	Sub-categroy definition	Anchor example
Practice organisation	Pertains to preparation and changes in practice organisation.	“We have tried to organise the workflows a bit, to structure them so that patients have fewer waiting times. That means we did not order quite as closely.” (Michael Haase, cardiologist)
Personal protective equipment (PPE)	Pertains to experiences in the procurement and work with PPE.	“Yes, well, we have all the hygiene standards, that we can have, we have done. It starts with disinfectants, it starts with the fact that the nurses in the registration area work with face masks, that we have placed a spitting protection, that we only allow a very small number of patients into the practice. I also work here permanently with a face mask.” (Steffen Hagel, family physician)
Alternative ways of healhcare provision	Pertains to changes in the provision of health care.	"Of course, it was also challenging, I talked to the patients on the phone a lot during that time. [...], I did telephone consultations." (Georg Hassel, cardiologist)
Social tension	Pertains to the importance of social relationships to patients and colleagues.	“At the very beginning, I spoke against it. I have stopped doing that in the meantime, because the people who are speakers have an entrenched opinion and they do not listen to you. That often ends up in discussions that steal my time and simply - the patients become aggressive. So then, I always try to say that everyone is allowed to have his opinion. [...] Especially when they tell me there is no Corona and we have never seen a positive one, I prove it by saying “I do”.” (Claudia Müller, non-physician assistant)
Management of pandemic information	Pertains to the challenges of managing the large amount of pandemic-related information.	“I am informed. (laughs) You are so bombarded with news and scare stories, so you do not have to make any special effort. Instead, it just comes flooding in, yes.” (Steffen Hagel, family physician)
Burden	Pertains to the importance of emotional and physical distress in everyday work.	“I think that is exhausting. For the patients, of course, but it is also exhausting for me, yes. So again and again you have to say: “Please remember, there are not allowed to be so many patients in the waiting room”. When I open the door to bring in the next one, there are 10 in the small waiting room. Then I repeat that. I always have to repeat, repeat, repeat everything, then the patients feel patronized [...] And sometimes - on Friday - you often get tired of it. […] That is why I still do - I still love our work. And I love the life in the practice. And I like the patients for the most part. Yes, so it is a special situation for everyone.” (Claudia Müller, non-physician assistant)

#### Practice organisation

Practice organisation seemed to be a key aspect of working conditions. Participants described being unprepared for a pandemic, *“[…] because this was a completely new situation […] you had to find a way to get through it sensibly" (Georg Hassel, cardiologist).* Decision-making was situational. Their aim was to develop a customized practice organisation as quickly as possible. In addition to PPE (face masks and protective gowns), participants reported about perspex walls at the reception and the physician’s workplace, capacity reduction in waiting rooms and outdoor waiting facilities, as well as (new) structured workflows, e.g. elective and infection consultation hours, and regular personal screenings.

#### Personal protective equipment

Personal protective equipment as a measure for infection protection and control seemed to have gained importance for the organisation in practice, and thus also for working conditions. Participants reported about price increases and poor quality of PPE due to higher demand. *“A huge problem in March and April [...] was the [...] protective equipment. When I begged my pharmacist for a bottle of disinfectant, more, and he sold me one for ten euros, yes, 500 milliliters. On the other hand, protective masks, [...] it was sometimes the case that the number was sufficient [...] but the quality was so shitty, so bad that every-, I (had) to throw away every second mask because it tears off my ear, yes. [...] I (got) blood pressure every time, yes, when you have something like that" (Georg Hassel, cardiologist).*

Furthermore, they established *“concept(s) relatively early”* that required *“wearing of masks and FFP masks and reduced [...] actual contact with patients also as much as possible" (Franz Schulze, cardiologist).*

#### Alternative ways of care provision

The participants described changes in the provision of healthcare such as telephone consultations, additional home visits, and arrangements with nursing providers or even consultations *“at the window and through the window" (Volker Heinze, family physician).*

*“I talked to the patients on the phone a lot during that time. […] did telephone consultations. I did not work less" (Georg Hassel, cardiologist).* Family physicians described how they used personal relationships with patients for health-related conversations outside the physician’s office to support the utilization of healthcare and avoid negative health-related effects. *“On the road with distance (laughs). Because I know a lot of them through the 30 years. […] my dear friend, your checkup was due" (Beate Beyer, family physician).*

#### Social tension

HCPs perceived changes in social relationships with patients and colleagues due to the pandemic. The participants reported a lack of clarity, a struggle of competencies and unclear responsibilities especially at the beginning of the pandemic. In addition, non-physician assistants in particular reported, that they had noticed changes in their interactions with patients. They described dissatisfaction, lack of understanding and displeasure - *“[...] yes, so this dichotomy and the-, there are rules everywhere, yes. I can’t just walk into the practice as usual. I first have to check whether there are still five people there or-. Exactly, kind of this togetherness. Yes, I would say the mood has changed a bit. […] But subliminally, I always notice that this lightheartedness, also in dealing with medical offices and like-minded people or with other patients who are waiting in there, has changed somehow" (Amelie Kohl, non-physician assistant).*

#### Management of pandemic information

All participants described challenges regard to the management of pandemic information *“because (for me), every week, there (was) new information, new approaches, new regulations regarding diagnostic procedures, billing" (Christoph Zobel, family physician)"* with a high daily workload to review the information and to implement the necessary measurements. In this context, the participants explained to prefer information sources they considered trustworthy, such as the Robert Koch Institute and professional associations. In order to manage the large amount of information, they also discussed distancing from the information overload and critical evaluation of information. The informal exchange with colleagues, e.g. during online training courses, was experienced as valuable.

#### Burden

The participants experienced emotional and physical burden in their working condition. Uncertainties due to the pandemic’s progression, as well as increased workload due to *“[…] almost every day […] new specifications from the Association of Statutory Health Insurance physicians" (Hannelore Betge, family physician)*, changes in practice organization, social tension, and worries and fears for themselves and their families seemed to have an emotional and physical impact on the HCPs.

*“[...] the patients are more annoyed or no longer quite so friendly or more introverted - in other words, this lightheartedness is not so there with some of them. [ …] I have to say, we are little affected up here with this whole situation. Nevertheless, it is a burden-, you notice that the patients are occupied with it and that it burdens in some way" (Amelie Kohl, non-physician assistant).*

### Health-related outcomes

The category consisted of three sub-categories and described the perceived short- and long-term effects as well as side effects on healthcare during the first year of the pandemic. The sub-categories along with brief definition and anchor example presented in Table 5:

**Table 5:** Health-related outcomes **-** sub-categories, brief definition and anchor example

Sub-category	Sub-categroy definition	Anchor example
Deficits in monitoring	Pertains to deficits in routine monitoring.	“[…] we have of course a deficit in the control, the routine monitoring of the patients like blood pressure, cholesterol controls, since now many patients [...] did not come to the blood test or did not come to the ECG, [...] Accordingly patients have withdrawn themselves from the routine monitoring and we have there certainly also catch-up need.” (Volker Heinze, family physician)
Health disadvantages	Pertains to the discourse on health disadvantages.	"We have had some things where we would have liked to respond sooner. Where the patients just did not come." (Martina Stünznagel, family physician)
Psychological impacts	Pertains to psychological consequences.	“Yes, psychological, of course. So psychological effects - depression in any case, yes. [...], where people are branded as potential virus carriers, yes. Whom do I infect- do I infect my grandmother or I now my grandchildren for example. [...] It is quite clear that this has psychological consequences. So I’m assuming - we’ll see - that the suicide rate will also rise in the coming months, i.e. winter depression plus Covid, in any case.” (Helmut Hosang, cardiologist)

#### Deficits in monitoring

The participants reported *“deficit in the monitoring, the routine monitoring of patients such as blood pressure, cholesterol checks, because now patients (did) not come for a quarter to get their blood drawn or (did) not come for an ECG" (Volker Heinze, family physician).* However, the impact was assessed differently.

#### Health disadvantages

Family physicians in particular reported that they did not perceive any pandemic-related health disadvantages for their patients. *"We don’t notice much of a difference" (Otto Pieger, family physician).* They described that patients had made decisions to use healthcare services consciously and, as a result, had fewer conversations about trivial matters than usual*.* In contrast to this, participants associated adverse events (e.g. hospital admissions with acute conditions or death, for example, due to myocardial infarction) with a decrease in consultations and follow-up visits, as well as concerns and fears about utilization of healthcare. Some patients with *“[…] problems […] sat it all out at home. To what extent this (had) worsen now, for the individual clinical picture, I do not know now. However, I can imagine that one or the other was simply postponed and the problems intensified" (Marion Stittich, family physician).*

#### Psychological impacts

All participants reported an increase in psychological and psychosomatic complaints, especially uncertainties, pressure, anxiety, and *“[…] depression in any case”*, which they considered to be effects of the pandemic. They described that patients were afraid to be infected in the practice. *“Yes, some of them just went to the prescription at the front - that was often because they were afraid to stay here in the practice for a long time. [...] They then get a prescription at the front and say: “next time, when Corona is over or so, we will measure blood pressure again and so on* *" (Karl Walther, family physician).*

## Discussion

Our findings from Saxony-Anhalt demonstrated how HCPs in rural areas maintained outpatient care during the COVID-19-pandemic. The outpatient HCPs immediately went to great lengths to re-organise and adapt standard procedures in their practices [40] and managed challenges with protective equipment and pandemic information. Some also used alternative forms of the physician-patient consultations (window and open-air practices) [41] and personal contacts with patients to support utilization of care. Social tension and mental burden seemed to interact with a perceived lack of preparedness, the pandemic-related changes as well as utilization of care. The toll of the crisis has been heavy on healthcare workers [42, 43]. In line with previous studies, outpatient HCPs described a lack of preparedness and impacts on providing outpatient care [28, 41, 44–46, 47, 48, 49, 50]. Although testing for SARS-CoV-2 infection was established nationwide early on, participants rarely discussed this in our study. Chronically ill and elderly people in particular make up a large proportion of patients in German outpatient care. They are dependent on regular medical consultations and have a high risk for severe disease courses, once infected with Covid-19 [51]. Fear of infection with the SARS-CoV-2 virus had led to a delay in necessary visits to medical care [52–54]. Previous studies showed changes in clinical work e.g. through re-organizing practice and the using alternative consultations [28, 40, 41, 44, 47–49, 55, 56]. We observed a switch towards telephone consultations, neither to video consultations. In line with Due et al., 2021 alternative consultation forms seemed to be context-bound, which again influence their willingness to use these alternatives [56]. In contrast to primary care physicians, the secondary care physicians reported less of a decline in healthcare utilization during the first wave of the pandemic. This could be related to the difficulties in accessing specialists in rural areas. Windak et al. reported that Family Physicians experienced that acute care was compromised, both by changed focus on respiratory assessment and triage and by the fact that patients consulted them less frequently for non-COVID-19 problems [57]. Monitoring visits were postponed or canceled. Consultations for complaints like low back pain, gastrointestinal complaints, vertigo or fatigue and services like housecalls/calls at nursing homes, wound treatments, pain therapy or screening examinations for the early detection of chronic diseases were particularly affected [54]. Some HCPs reported concerns of collateral damage to the health of the population due to abandoned or postponed routine care [58–60]. Furthermore, HCPs experienced effects on patients’ psychological well-being. As mental resilience decreased, symptoms of depression, anxiety, and somatization increased [61].

### Strengths and limitations

Our study provides insights into how outpatient HCPs in a rural area in Germany experienced their work during the COVID-19. In a time of intense workload, we succeeded in recruiting 28 healthcare professionals from Saxony-Anhalt, Germany. It has to be mentioned that the interviews took place after the first COVID-19 wave in Germany, which was controlled by a strict lockdown strategy resulting in a relatively low COVID-19 case count in many districts [62]. Since the interviews were conducted by telephone and in part under perceived time pressure for the participants, it was sometimes difficult to build trust. Furthermore, it might have influenced the questioning behavior of the interviewers as well as the answering behavior of the participants. The subsequent translation of the research results and quotations carries the risk of losing or alienating the meaning [63]. With only women conducting all interviews, we cannot exclude gender dynamics in the interviews [64]. In addition, differences in qualitative research expertise or prior professional experience in healthcare might have influenced data collection and analysis, too. Our sample included only HCP from Saxony-Anhalt. Rural areas are nuanced, and each has unique variable pressures, which may alter perceptions: in particular, the number of people infected with Covid-19, the shortage of primary care physicians [8] and higher prevalence of chronic conditions (diabetes, hypertension, and heart failure) [11, 14, 65] and socioeconomic factors [66]. Future work could summarize continue to explore the results of HCP studies in rural areas related to the experience of the COVID-19 pandemic.

## Conclusion

The COVID-19-pandemic has highlighted the challenges in healthcare rural areas in a particular way. In the first year of the pandemic, outpatient HCPs experienced impacts on access to and utilization of healthcare, on working conditions and health-related outcomes in their patients. Outpatient physicians provided access to health care. Within a short time, HCPs re-oranganized their clinical work, managed challenges with PPE and pandemic-related information. We determined psychological impacts and mental burden among all those involved in the outpatient care, including patients. These influenced the perception of the daily practice and, in the case of patients, the utilization of care. Our study suggests that HCPs in rural areas should be supported in terms of healthcare infrastructure, and strategies for working conditions.

## Supplementary Information

**Additional file 1.**

## Data Availability

The datasets used and/or analysed during the current study are available from the corresponding author on reasonable request.

## Abbreviations

COVID-19: Corona Virus Disease 2019
FP: Family physician
HCP: Healthcare professional
KARLA: KARdiologische LandAssistenz (Cardiology support in rural areas)
PPE: Personal protective equipment
