# Supplementary material for: Healthcare professionals’ perceptions of impacts of the Covid-19-pandemic on outpatient care in rural areas: a qualitative study
Source: BMC Health Serv Res. 2021 Dec 2;21:1298. doi: 10.1186/s12913-021-07261-y (PMC8638652; doi:10.1186/s12913-021-07261-y)
Supplement: Supplementary file 1 — Additional file 1. [file 12913_2021_7261_MOESM1_ESM.docx]

**Interview guide**

- How do you experience the care of patients with chronic heart diseases in your daily work?
- What outpatient services are available regionally for patients with chronic heart diseases?
- How do you cooperate with medical colleagues in primary, secondary and tertiary care to ensure adequate treatment for patients with chronic heart diseases?
- What do you consider to be the needs for care and support in the everyday life of patients with chronic heart diseases?
- Which challenges do you see in order to cover the care and support needs of patients with chronic heart diseases in the medium term?
- In which aspects would you like to receive support in treatment and support of cardiological patients?
- How do you currently experience the quality of cardiology care from your professional perspective?
- How do you see your role in outpatient cardiology care?
- What is the role of economic considerations in the provision of health care services?
- How do you experience your work during the COVID 19 pandemic?
- For what reasons have, your patients visited your practice in recent weeks.
- How are you currently manage to get reliable information?
- Which health effects do you perceive in your patients due to COVID-19?
